# Authors’ response to the letter to the editor entitled: Co-circulation of dengue, chikungunya, and Zika viruses and cross-protection

**DOI:** 10.26633/RPSP.2019.77

**Published:** 2019-08-22

**Authors:** Alejandro Rico-Mendoza, Alexandra Porras-Ramírez, Aileen Chang, Liliana Encinales, Rebecca Lynch

**Affiliations:** 1 Grupo de Medicina Comunitaria y Salud Colectiva Grupo de Medicina Comunitaria y Salud Colectiva Universidad El Bosque Bogotá Colombia Grupo de Medicina Comunitaria y Salud Colectiva, Universidad El Bosque, Bogotá, Colombia.; 2 Department of Medicine Department of Medicine the George Washington University Washington, D.C. United States of America Department of Medicine, the George Washington University, Washington, D.C., United States of America.; 3 Allied Research Society Allied Research Society Barranquilla Colombia Allied Research Society, Barranquilla, Colombia.; 4 Department of Microbiology, Immunology, and Tropical Medicine Department of Microbiology, Immunology, and Tropical Medicine the George Washington University Washington, D.C. United States of America Department of Microbiology, Immunology, and Tropical Medicine, the George Washington University, Washington, D.C., United States of America.

To the editor:

Dengue, zika, and chikungunya outbreaks in Central and South America countries have presented significant challenges related to their prevention and control. From the virologic point of view, the possibility has been raised that the co-circulation of the three viruses could generate cross-protection between the three alphaviruses.

In order to discuss this hypothesis it must be taken into account that Zika, dengue (DENV) and chikungunya viruses are closely related flaviviruses, with identical urban transmission and some immune interactions (1). Also, it is known that secondary DENV infections may be more severe than primary infections due to the antibody-dependent immune response (i.e., heterotypic sub-neutralizing antibodies that increase virus entry into poorly susceptible cells) (2,3).

In addition, the recent introduction of Zika and chikungunya viruses in the Americas and the large-scale exposure of a uniformly unexposed population could affect subsequent transmission of dengue virus. This hypothesis has not been tested, largely because insufficient epidemiological data are available for the affected sites. However, in Salvador, Brazil, after the zika outbreak there was a significant decrease in the frequency of dengue cases (4). A similar situation was observed in Colombia, where the decrease in dengue cases following the zika and chikungunya outbreaks went from 334.1 cases per 100 000 people in 2015 to 90.7 cases per 100 000 in 2017 and 173,1 cases per 100 000 in 2018 (5). Although temporary associations do not prove causation, the strength and consistency of the observations suggest that infections with Zika virus and chikungunya virus could induce cross-protective immunity against dengue. Prospective studies are needed to fully assess the risk of dengue infection after exposure to Zika and chikungunya viruses and to determine whether the supposed cross-protection is long-lasting. Although observations support this hypothesis, the potential direct implications of this hypothesis for epidemiological surveillance, immunological research on pathogenesis and vaccine development require additional studies.

## Declaration.

The opinions expressed in this manuscript are the responsibility of the authors and do not necessarily reflect the criteria or policy of the RPSP / PAJPH and/or PAHO.

This reply refers to the letter available at: https://doi.org/10.26633/RPSP.2019.75
